# A mouse model of prostate cancer bone metastasis in a syngeneic immunocompetent host

**DOI:** 10.18632/oncotarget.27317

**Published:** 2019-12-03

**Authors:** Brian W. Simons, Vishal Kothari, Benjamin Benzon, Kamyar Ghabili, Robert Hughes, Jelani C. Zarif, Ashley E. Ross, Paula J. Hurley, Edward M. Schaeffer

**Affiliations:** ^1^The Brady Urological Institute, Department of Urology, Johns Hopkins University School of Medicine, Baltimore, MD, USA; ^2^Department of Molecular and Comparative Pathobiology, Johns Hopkins University School of Medicine, Baltimore, MD, USA; ^3^Department of Oncology, Johns Hopkins University School of Medicine, Baltimore, MD, USA; ^4^Department of Urology, Feinberg School of Medicine, Northwestern University, Chicago, IL, USA

**Keywords:** prostate cancer, bone metastasis, murine model

## Abstract

We report the establishment of B6CaP, an allograft tumor line from a Hi-Myc transgenic mouse that had been backcrossed onto C57BL/6J background. This tumor line grows subcutaneously in wildtype C57BL/6J immunocompetent mice, expresses AR, and has a luminal cytokeratin profile. When digested into single cells and injected via intracardiac injection, B6CaP produces metastatic widespread metastases including frequent bone lesions. Metastatic lesions occur most often in the femur, spine, and skull, and have a mixed osteolytic/osteoblastic phenotype. B6CaP allografts are androgen dependent, and regress after castration. However, castration resistant tumors regrow after 4–6 months and can be maintained as androgen-independent clones. This is the first example of a prostate-derived tumor line that shows frequent metastasis to bone and grows in an immunocompetent host, making this model useful for studying mechanisms of bone metastasis and tumor immune response.

## INTRODUCTION

Prostate cancer (PCa) is the most common non-cutaneous cancer in the United States, and is the third leading cause of cancer-related deaths in men [1]. Advanced PCa accounts for more than 80% prostate cancer-related deaths [2] due to frequent skeletal metastasis. Thus, a mechanistic understanding of metastatic events and development of new, effective therapies are critically required to treat and prevent PCa bone metastases. Animal models of cancer allow the study of molecular mechanisms of disease progression and test new treatments across the disease sites; but an uncommon occurrence of spontaneous prostate cancer in mice [3] and a lack of animal model systems that closely recapitulate the human PCa hampers the mechanistic understanding of metastatic progression and development of effective treatments for advanced PCa.

While xenografts of human cell lines in immunodeficient mice remain the most commonly used models, PCa cell lines rarely metastasize from subcutaneous grafts, with an exception of a few cell lines that metastasize when injected orthotopically [4]. This limits the studies of bone metastasis to a small number of cell lines or direct injection of cells into bone, usually tibia. All xenograft models require immunodeficient host mice, which not only precludes the analysis of tumor immune response and development of immunotherapies, but also provides insufficient details on metastasis since the metastasis takes place in an immune-deficient environment in these models. Additionally, species differences can interfere with interaction between human tumor cells and mouse stroma. To overcome these problems, numerous genetically engineered mouse models of PCa have been developed over the years (reviewed in [5]) including the TRAMP model [6] that displays PCa metastases to distant organs such as lung, but rarely to bones, a feature consistent in other transgenic mouse models as well (reviewed in [5]). Intriguingly, the metastatic cells in many transgenic mouse models including TRAMP are found to be of neuroendocrine origin [7–9]. Given that majority of clinical cases of prostate cancers are adenocarcinoma with limited neuroendocrine differentiation, it is critical to develop animal model systems that closely recapitulate human prostate adenocarcinoma and subsequent metastatic events seen in the clinics.

Here we describe the generation of B6CaP, an allograft tumor line obtained by backcrossing Hi-Myc transgenic mice with C57BL/6J. B6CaP cells can grow subcutaneously in immunocompetent wildtype C57BL/6J mice, expresses AR, and shows a cytokeratin profile of luminal PCa. Intracardiac injection of B6CaP results in frequent skeletal metastases, making it an excellent pre-clinical model to study the mechanisms of metastasis in PCa. Further, B6CaP allografts can be grown either as androgen-dependent or as castration-resistant lines. To our knowledge, B6CaP is the first-in-field prostate-derived tumor line that shows frequent skeletal metastases and can be grown in an immunocompetent host, making it possible to study the mechanisms of bone metastasis and tumor immune response in an immunocompetent background.

## RESULTS

### B6CaP allograft recapitulates luminal epithelial phenotype of autochthonous Myc-driven prostate cancer

**Figure 1:**
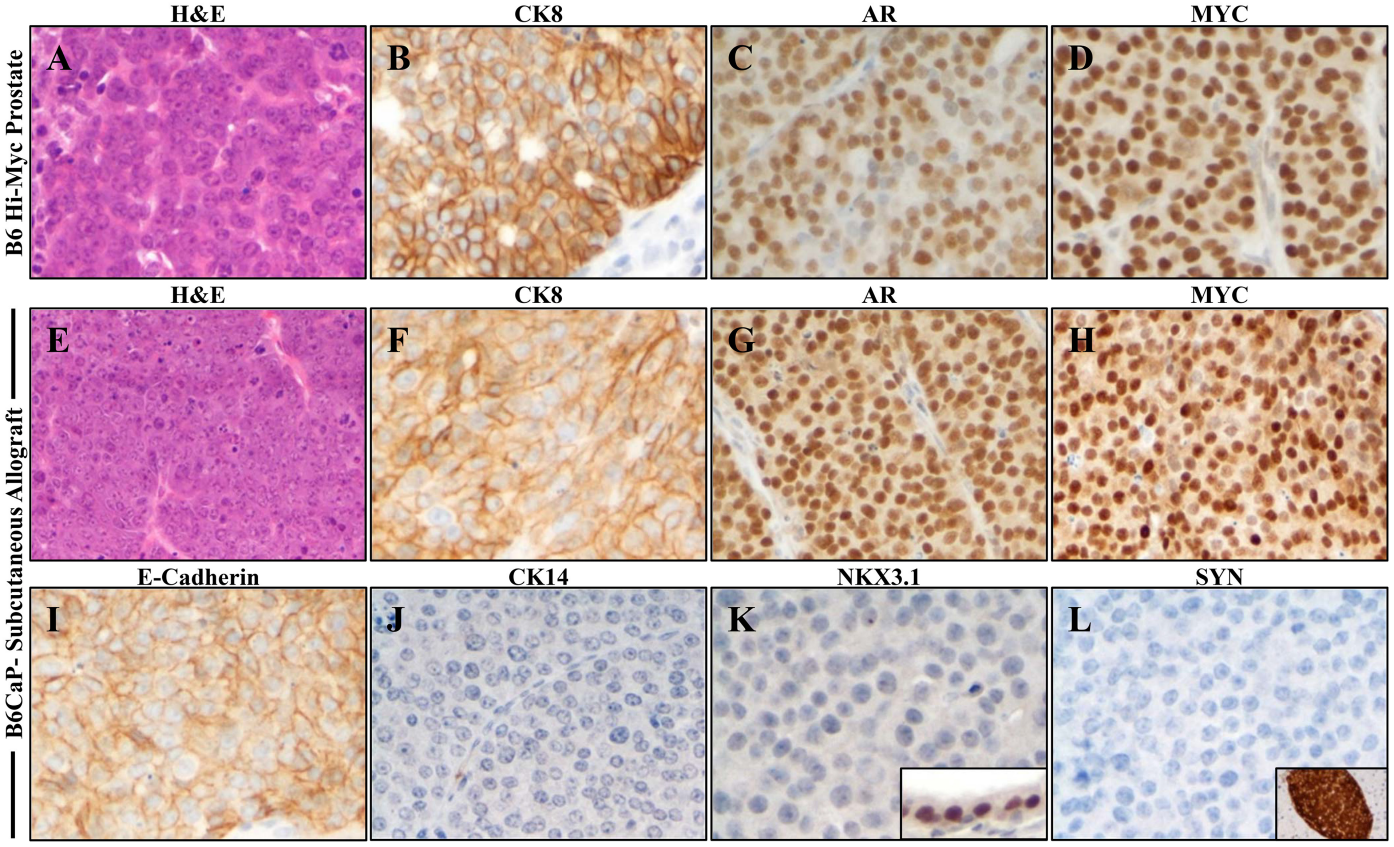
B6CaP allograft recapitulates luminal epithelial phenotype of autochthonous Myc-driven prostate cancer. (**A–D**) Autochthonous tumor from a C57BL/5J Hi-Myc mouse expresses luminal cytokeratin 8 (CK8), and high levels of nuclear Androgen Receptor (AR), and MYC. (**E–H**) Subcutaneous allograft B6CaP expresses similar levels of CK8, AR, and MYC. (**I–L**) B6CaP maintains E-Cadherin expression, lacks expression of cytokeratin 14 (CK14), NKX3.1, and Synaptophysin. Insets indicate positive control stains in normal prostate (K) and pancreatic islet (L).

The Hi-Myc transgenic mouse model of prostate cancer was generated on an FVB/N inbred background [10]. Although the Hi-Myc mice showed the development of prostatic intraepithelial neoplasia (PIN) and its progression to invasive carcinoma, metastasis was absent in this model [10]. Since most targeted mutations are now generated or are available on a C56BL/6 background, we crossed the Hi-Myc line to the C57BL/6J background for more than ten generations. SNP analysis (Genome Scanning Service, Jackson Laboratory) of the resulting line confirmed 100% identity with C57BL/6J background (Supplementary Table 1). Consistent with previous reports, mice on this background showed a longer latency to prostate cancer, with invasive lesions arising after 15 months of age, compared to 6 months for FVB background Hi-Myc mice [11]. Similar to the FVB background, metastases in this model were rare, but widespread metastatic lesions were identified in two 17–18-month-old mice. Tissue from a lymph node metastasis from one mouse was dissociated and injected subcutaneously into a C57BL/6J mouse to generate a serially transplantable allograft line, B6CaP. When compared to advanced but localized prostate tumors in B6 Hi-Myc mice (Figure 1), B6CaP allografts maintained a similar phenotype of luminal cytokeratin expression (CK8), strong androgen receptor (AR) expression, and high levels of MYC expression from the Hi-Myc transgene [10] (Figure 1A–1H). The allografts also maintained expression of the epithelial marker E-Cadherin, and were negative for basal cytokeratins (CK14) and markers of neuroendocrine differentiation such as Synaptophysin (SYN) (Figure 1I, 1J). Similar to advanced prostate tumors in Hi-Myc mice, B6CaP allografts did not express NKX3.1 [10, 12] (Figure 1K, 1L). B6CaP allografts maintained similar expression patterns over multiple passages (currently passage 5), and images in Figure 1 represent typical results.

### Immune profile of B6CaP shows infiltration of immune-regulatory cells

**Figure 2:**
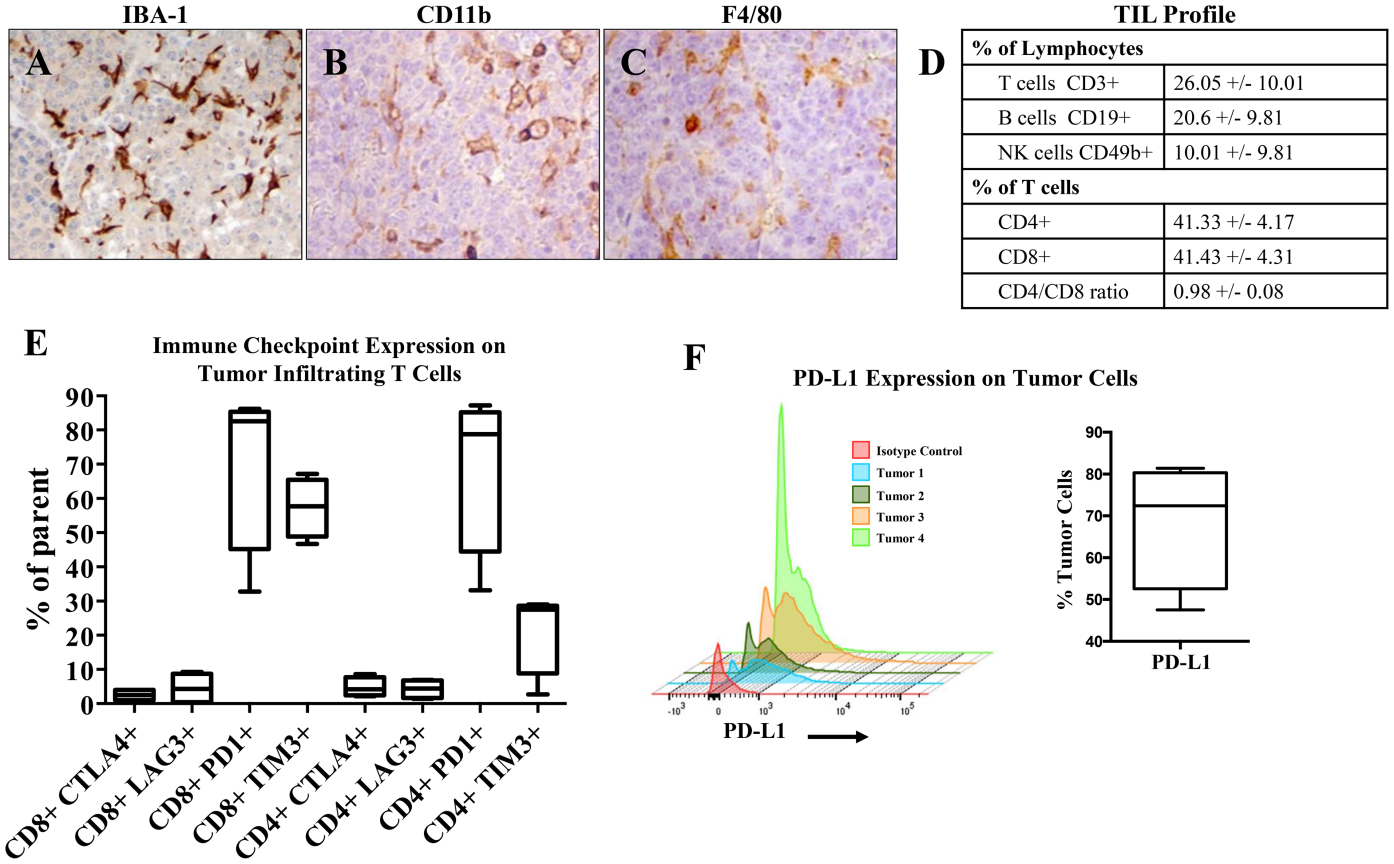
Immune profile of B6CaP. (**A, B**) Immunohistochemistry for IBA-1 (A), CD11b (B), and F4/80 (**C**) indicate high levels of infiltration by macrophage/myeloid cell types. (**D**) Tumor infiltrating lymphocyte (TIL) populations characterized by flow cytometry of dissociated allografts (mean +/– standard deviation). (**E**) Immune checkpoint expression of tumor infiltrating T cells. (**F**) PD-L1 expression of dissociated tumor cells.

We next sought to characterize the immune infiltrate of B6CaP subcutaneous allografts. Immunohistochemistry demonstrated infiltration of numerous macrophages (Figure 2A–2C). Further analysis of lymphocyte populations by flow cytometry (*n* = 4 third passage B6CaP tumors grown in parallel) indicated abundant infiltration of CD3+ T cells, CD19+ B cells, and CD49b+ NK cells (Figure 2D, Supplementary Figure 1). Since immunotherapy regimens most commonly employ monoclonal antibodies targeting regulatory checkpoints [13], we determined the expression levels of four immune checkpoints (CTLA4, LAG3, PD1, and TIM3) on tumor infiltrating T cells, and PD-L1 expression on B6CaP tumor cells. Infiltrating T cells, especially CD8+ T cells, often expressed PD-1 and TIM3, and tumor cells were frequently positive for PD-L1 (Figure 2E–2F, Supplementary Figure 2).

### Growth and metastasis profile of B6CaP cells

In order to determine the growth and metastasis profile of B6CaP cells, we digested freshly resected allografts into single cell suspensions and implanted them into immunocompetent C57BL/6J mice by multiple routes (Table 1). When implanted as flank subcutaneous allografts, tumors formed in 48/50 (96%) mice. The mice were euthanized for tissue collection when the tumors reached 1.5 cm diameter. At this timepoint, 11/48 (23%) mice had metastatic lesions in the lungs. Due to a rapid growth of the subcutaneous tumors, the survival of injected mice was limited to approximately 6 weeks. In order to allow a longer time period for metastases to develop, established subcutaneous xenografts were surgically resected when the tumors were 1 cm in diameter and the mice were followed for an additional 8- or 16-weeks post resection. At these later time points, metastases were identified in 21% and 67% of mice, respectively. Orthotopic injection of tumor cells into the anterior prostate also showed metastatic potential, with 60% of animals showing metastasis to lungs (Table 1), and abdominal lymph node metastases present in 4/10 (40%) of mice. Intravenous inoculation of B6CaP cells resulted in formation of metastatic foci in multiple sites, including bone, but growth of numerous lung metastases often resulted in early euthanasia. Intravenous injection of 1 × 10^5 B6CaP cells resulted in formation of lung tumors in 14/21 mice (67%). By comparison, injection of 1 × 10^5 cells from the Myc-CaP cell line into syngeneic FVB/N mice resulted in formation of lung metastases in significantly fewer mice (3/11, 27%, *p* = 0.034).

**Table 1:** Metastasis profile of B6CaP allografts

**Delivery**	**Local Growth**	**Lung Mets**	**Bone Mets**
Subcutaneous	48/50 (96%)	11/48 (23%)	0/48 (0%)
Subcutaneous (organoid)	4/4 (100%)	2/4 (50%)	0/4 (0%)
SQ Resected 8 wks	N/A	4/19 (21%)	0/19 (0%)
SQ Resected 16 wks	N/A	8/12 (67%)	0/12 (0%)
Intravenous	N/A	14/21 (67%)	9/21 (43%)
Intracardiac	29/57 (51%)	26/57 (46%)	33/57 (58%)
Intracardiac (organoid)	0/4 (0%)	2/4 (50%)	2/4 (50%)
Orthotopic	10/10 (100%)	6/10 (60%)	0/10 (0%)
MycCaP Intravenous	N/A	3/11 (27%)	0/11 (0%)

IC Injection Metastases	Any Met	Any Bone	Skull	Femur	Spine	Adrenal	Seminal Vesicle
Number/% (*n* = 57)	44 (77%)	33 (58%)	30 (53%)	10 (18%)	15 (26%)	14 (25%)	7 (12%)
Other sites	Lung (26), Lymph Node (9), Eye (7), Epididymis (2), Brain (2), Spleen (2)						

B6CaP tumors were implanted by subcutaneous, intravenous, intracardiac, or orthotopic routes. Some subcutaneous tumors were resected when they reached 1 cm diameter. For comparison, MycCaP mice were injected into syngeneic mice by intravenous route. Columns represent number of mice with local growth at injection site, lung metastases, or bone metastases at necropsy. For mice with intracardiac B6CaP injections, lower panel indicates additional sites of metastases. “Any Met” refers to any distant metastasis, or growth at any site other than heart/pericardium.

**Figure 3:**
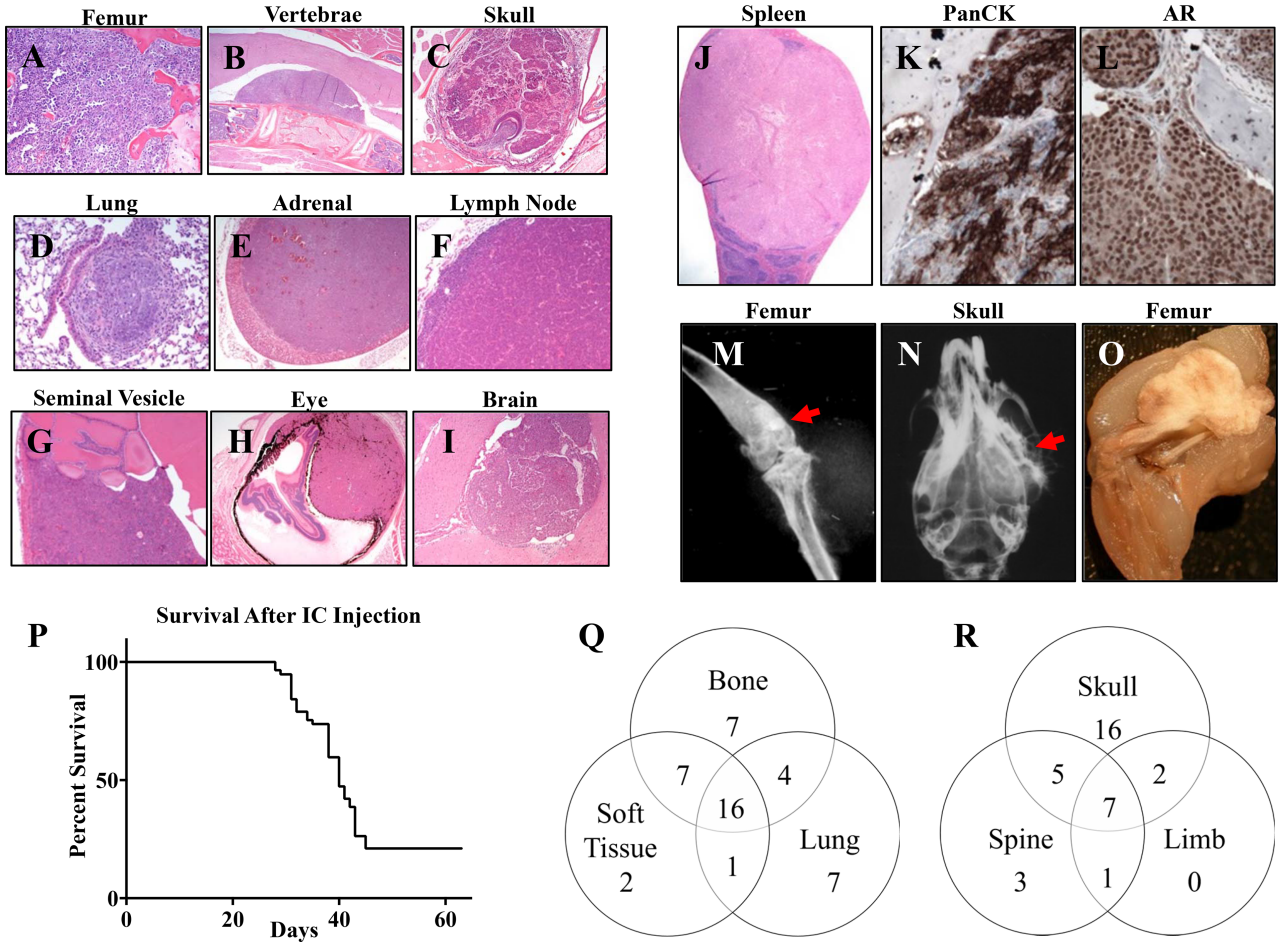
Intracardiac injection of B6CaP cells generates widespread bone and soft tissue metastases. Representative images (H&E) of metastases in (**A**) femur, (**B**) vertebrae with spinal cord compression, (**C**) skull/jaw (**D**) lung, (**E**) adrenal gland, (**F**) abdominal lymph node, (**G**) seminal vesicle, (**H**) Eye, (**I**) brain, and (**J**) spleen. (**K, L**) Immunohistochemistry for pan-cytokeratin (PanCK) and AR. Bone metastases generated osteoblastic (**M**) and osteolytic lesions (**N**). Bone lesions were occasionally large and exophytic (**O**). After intracardiac injection, median survival was 40 days with 21% (*n* = 12/57) of animals surviving to the 9-week endpoint (**P**). Venn diagrams representing the numbers of mice with metastases to bone, lung, or other non-lung soft tissue sites, total *N* = 44 (**Q**). Venn diagram representing the locations of bone metastases to skull, spine, or limbs, total *N* = 34 (**R**).

Intravascular inoculation of tumor cells, either intravenous injection via tail vein or intracardiac injection, generated widespread metastases with frequent bone metastases. However, since bone is the primary site of metastasis for PCa cells, we focused on characterizing the metastasis profile after intracardiac injection because the bone metastasis rate was higher. Mice were injected with 1 × 10^5 B6CaP cells by intracardiac route and followed for 9 weeks or until euthanized due to morbidity. Animals were examined for metastasis by gross inspection and histologic analysis of multiple organs including femur, skull, and spine after decalcification. Tumor growth at any location was noted in 96% of mice (55/57), including local growth in the heart, mediastinum, or skin over the injection site. Distant metastases were present in 44/57 mice (77%), with bone as the most common site of metastasis (Table 1 and Figure 3). Histological analysis showed frequent skeletal metastasis in femur, vertebrae and skull (Figure 3A–3C), as well as in soft tissues such as lung, adrenal gland, abdominal lymph node, seminal vesicle, spleen, eye and brain (Figure 3D–3J). Immunohistochemistry showed that these metastases retained epithelial differentiation (pan cytokeratin positive, Figure 3K), and expressed high levels of androgen receptor (AR) (Figure 3L). Radiographic examination of mice after intracardiac injection showed bone metastases in B6CaP model developed osteoblastic (Figure 3M) and osteolytic lesions (Figure 3N), and bone lesions were occasionally large and exophytic (Figure 3O). Overall, median survival after intracardiac injection was 40 days, with 21% of mice surviving to the 9-week endpoint (Figure 3P). The numbers of mice with metastasis to bone, lung, or other soft tissue sites as well the locations of bone metastasis to skull, spine and limbs are summarized in Figure 3Q and Figure 3R respectively.

### B6CaP is androgen-dependent but generates castration-resistant sublines with diverse phenotypes

**Figure 4:**
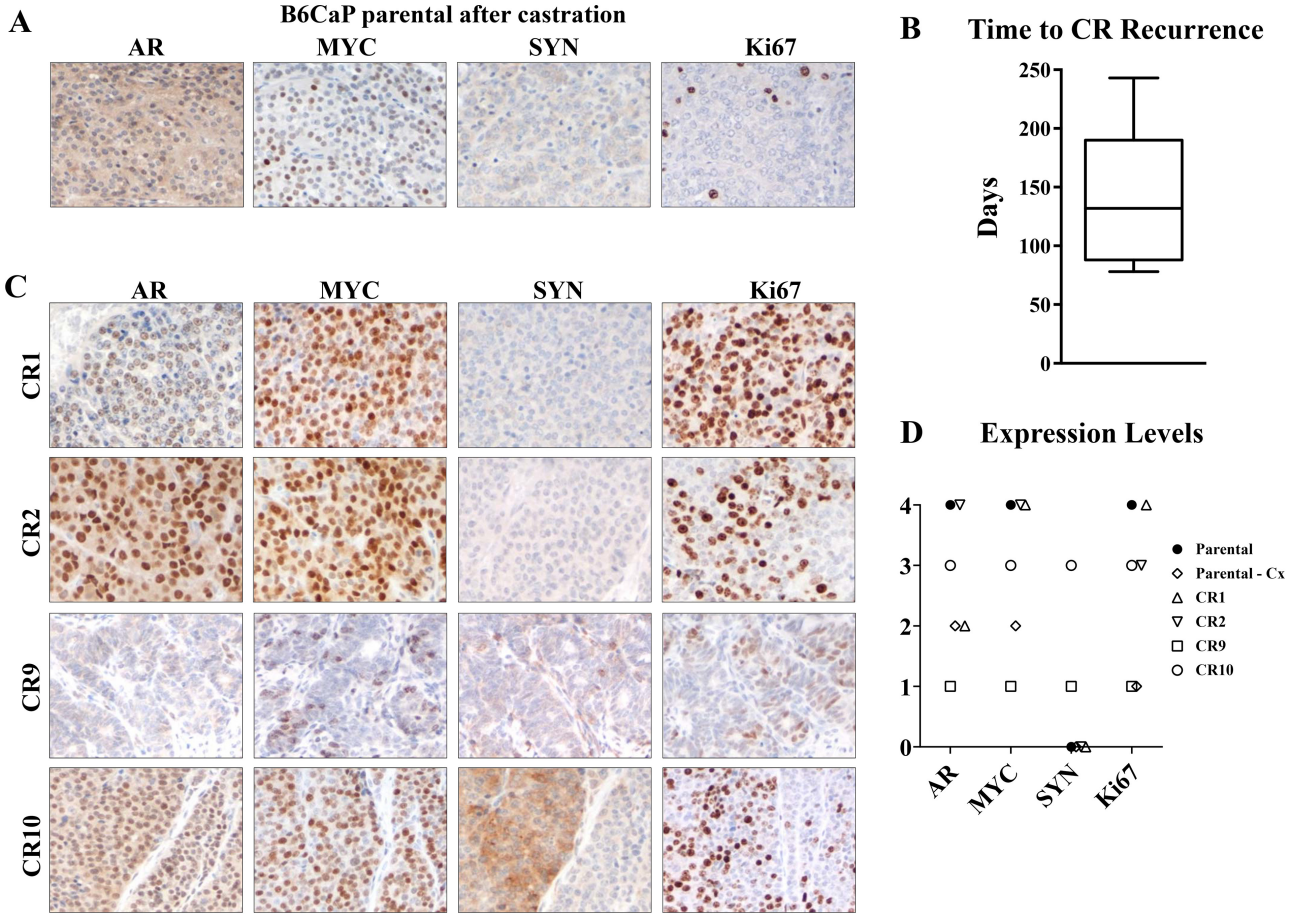
B6CaP generates castration resistant sublines with diverse phenotypes. B6CaP allografts partially regressed after castration and expressed low levels of cytoplasmic AR, MYC, and Ki67 but no synaptophysin (SYN) (**A**). B6CaP allografts partially regressed after castration, but eventually recurred as castration resistant tumors (CR). Mean time to recurrence was 140 days (**B**). Representative images of recurrent castrate resistant (CR) tumors (**C**). Increased proliferation (Ki67) and diverse expression of AR, MYC, SYN were found among the CR sublines (C). A representative slide from parental B6CaP, regressed B6CaP after castration, and each CR line was scored for expression of various markers (**D**).

In human patients with metastatic castration-sensitive prostate cancer, initial anti-androgen therapy is typically quite effective at reducing tumor burden [14]. After a period of dormancy, these tumors will recur as castrate-resistant prostate cancer. Many of these tumors retain high levels of AR activity through AR amplification or mutations [15], but a subset shows low or mosaic AR expression, or features of neuroendocrine differentiation [16–18]. Like bone metastasis, this pattern of strong response to androgen deprivation, followed by tumor dormancy and eventual recurrence as castrate-resistant tumors has been difficult to recapitulate in mouse models. To determine the effect of castration of B6CaP cells, tumor bearing mice were surgically castrated when tumors reached 1 cm in diameter. Castration induced rapid tumor regression until tumors stabilized in size at approximately 10% of the original tumor volume. At this point, tumor size remained stable for several months. Analysis of these tumors by immunohistochemistry (Figure 4A) showed low levels of diffuse, cytoplasmic AR staining, similar to AR expression in the fully regressed normal mouse prostate after castration. MYC expression, which is AR dependent in Hi-Myc mice, and proliferation (Ki67) were very low compared to B6CaP in intact mice (Figure 4A, Figure 1) while synaptophysin (SYN) expression (a marker of neuroendocrine differentiation) was not detected at this stage. Tumors began to regrow as castrate-resistant tumors after an average of 139 days (Figure 4B). At this stage, we resected palpable sized tumors and established multiple independent castrate-resistant (CR) sublines from the parental B6CaP which can be passaged in castrated mice. The phenotype of these cell lines varies with regard to expression of AR, MYC, and neuroendocrine markers. Most lines are similar to parental B6CaP and show moderate to high expression of nuclear AR, MYC and proliferation marker Ki67 (Figure 4C, CR1 and CR2). Interestingly, some CR sublines showed low AR and MYC expression (Figure 4C, CR9), while one subline showed evidence of neuroendocrine differentiation based on synaptophysin expression (Figure 4C–4D, CR10) that was low or absent in other lines.

### B6Cap can be propagated *in vivo* and *ex-vivo*

**Figure 5:**
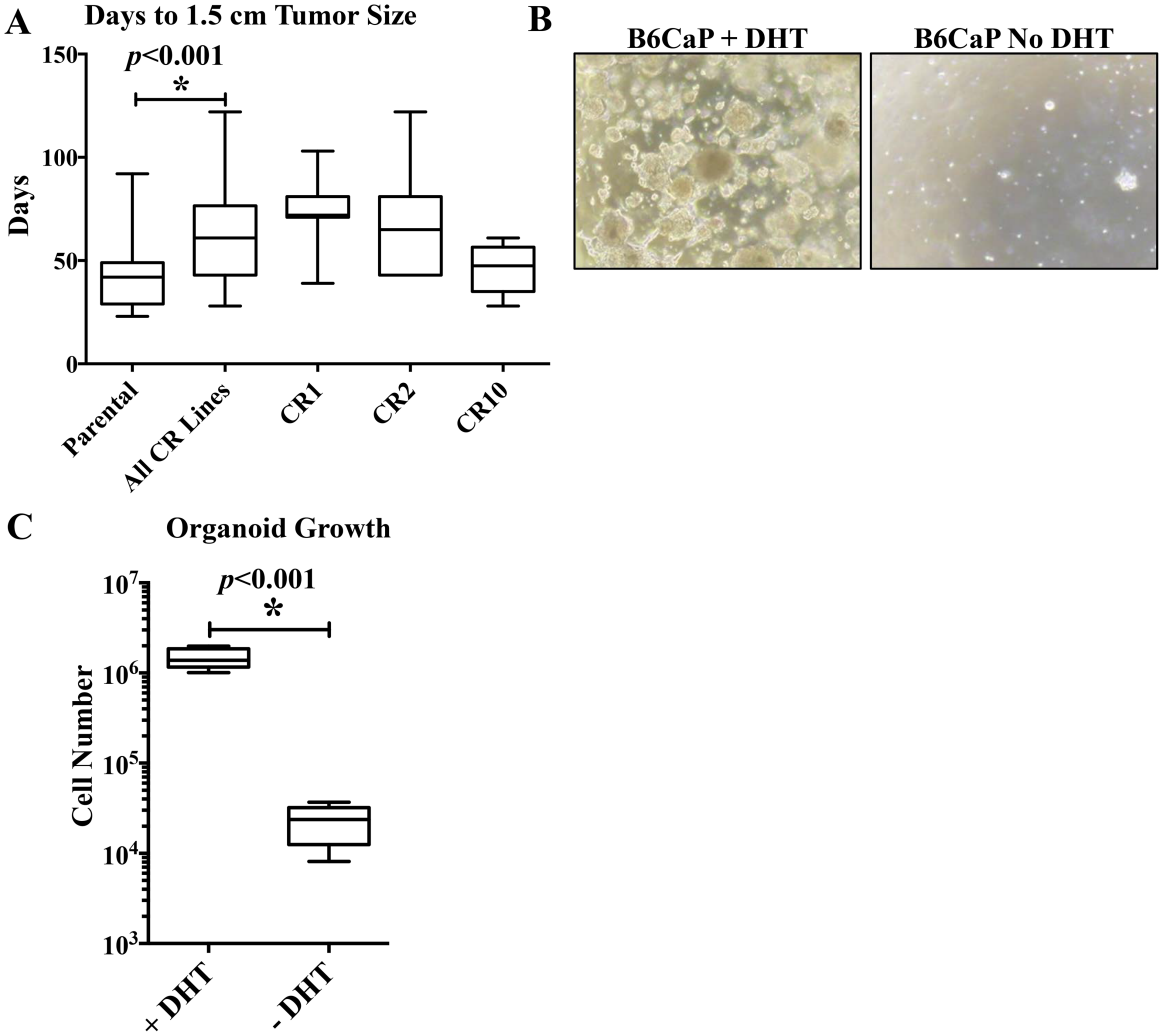
*in vivo* and *ex-vivo* growth kinetics of B6CaP. Growth rates of CR sublines in castrate hosts, measured as time from injection to 1.5 cm tumor size, was significantly slower than parental B6CaP cells in intact hosts (**A**, 42.9 vs 63.3 days, *p* < 0.001). Organoids retained androgen sensitivity *in vitro*, growing rapidly to form organoids/spheres in media containing DHT, but rarely forming small spheres without DHT (**B**). Three weeks after plating 10^5^ cells as organoids, cells in media containing DHT had expanded 10-fold, but total cell number had decreased in media without DHT (**C**).

We next tested the ability of CR sublines to grow in castrated hosts. We injected CR sublines in C57BL/6J hosts, and noted that overall, CR sublines in castrate hosts grew more slowly than B6CaP parental line in an intact host, taking 63 ± 4.6 days to reach 1.5 cm diameter versus 43 ± 2.4 days respectively (*p* < 0.001, Figure 5A). We have not yet developed an adherent cell line from the B6CaP allograft, However, allograft cells grow readily as organoids *in vitro* and remain androgen dependent (Figure 5B, 5C). Dissociated B6CaP cells were suspended in matrigel (1 × 10^5^ cells per 6-well plate) and cultured for 10 days with or without DHT. With androgen, cell number increased 10-fold, but viable cell number decreased in the absence of DHT. Preliminary attempts to recapitulate the metastatic phenotype from organoids suggest they retain a similar metastatic ability. After injecting 2 × 10^6 organoids subcutaneously, tumors grew in 100% of mice, and lung metastases were present at harvest in 2/4 (50%) of mice (Table 1). Intracardiac injection of 1 × 10^5 organoids resulted in bone metastasis in 2/4 (50%) of mice (Table 1). Additional studies will be needed to determine if the pattern and rate of metastasis are similar from organoid derived cells.

## DISCUSSION

Metastasis of prostate cancer is a multistep phenomenon that involves complex processes such as neovascularization, acquisition of invasiveness, invasion, intravasation, circulation of tumor cells in the blood, organ homing, dormancy, and secondary growth or metastasis. Bone, the primary site for PCa metastasis, adds another layer of complexity to this multistep process by influencing prostate cancer cells via an array of stimuli originating from hematopoietic cells, cytokines and bone remodeling activities [19]. Further, the cross talk between PCa and immune cells is crucial to the metastatic spread of PCa cells [20]. The prognosis for metastatic PCa remains poor despite significant advances in immunotherapies and second generation anti-androgen therapies [21]. Hence, a deeper understanding of molecular mechanisms of metastasis is indispensable to develop new treatment strategies and to achieve better clinical outcomes. Unfortunately, till date, no mouse model exists to study the skeletal metastasis of PCa cells in an immune-competent environment. To overcome this limitation, we developed an allograft tumor line, B6CaP, that facilitates the skeletal metastasis of PCa in the backdrop of a fully functional immune system.

We chose C57Bl/6J background to generate the allograft line B6CaP, since a majority of the targeted mutations are now available on this background. Hi-Myc mice [10], which show the development of PIN and invasive carcinoma, but not metastasis, were crossed with immunocompetent C57BL/6J mice. Myc is one of many oncogenes that play a role in prostate carcinogenesis. To date, we have not determined the expression or mutation status of PTEN in B6CaP cell lines, which is commonly altered in metastatic human prostate cancer [22]. We harvested PCa cells from the lymph node metastasis and injected these cells subcutaneously to establish a serially transplantable allograft line, B6CaP. Molecular characterization showed that this line expressed hallmarks of adenocarcinoma and is positive for AR and luminal cytokeratin (CK8). Markers of neuroendocrine differentiation were absent in this line. Like all cell lines, B6CaP was established from a single, rare, metastatic event in one mouse. Although not derived from a single cell like clonal cell lines, there may be limited heterogeneity among cells.

Hormone-naïve prostate cancer regresses upon androgen-deprivation (castration), however, it eventually regrows to a castration-resistant phenotype. Similar to the observations made in clinics, B6CaP allografts regressed after castration, but eventually regrew as castration-resistant (CR) tumors. These castration-resistant tumors could be maintained as CR lines in castrated hosts as well as in organoid forms, albeit with a slower growth rate compared to their hormone-sensitive counterparts. Since these observations closely recapitulate the clinical conditions, the model system described here could allow for studies to understand the molecular mechanisms underlying the development of castration-resistance.

Characterization of the immune infiltrate of B6CaP line demonstrated infiltration of multiple immune-regulator cells such as macrophages, CD3+ T cells, CD19+ B cells, and CD49b+ NK cells indicating that B6CaP can grow on an immune-competent background. Of note, the Hi-Myc transgene contains sequence for expression of the FLAG epitope. It is unclear if this epitope is expressed in Hi-Myc mice or in B6CaP cell line, but expression could affect the immune response. Interestingly, infiltrating immune cells were found positive for PD-1 and TIM3 and B6CaP cells were positive for PD-L1. These molecular features may facilitate immune checkpoint studies in a fully functional immune background, and may help developing effective immunotherapies for prostate cancer either alone or in combination with other therapies. Further, our model may allow researchers to study the acquired mechanisms of resistance to various immune therapies.

One of the major limitations of most current mouse models is the lack of metastasis of PCa cells to bone. While several human prostate cancer cell lines will metastastasize to bone after intracardiac injection, bone metastases are very rare or non-existent in most autochthonous models. Mouse cell lines and allografts also show limited bone metastasis with the exception of RM1(BM), a cell line derived from embryonic urogenital sinus epithelium expressing multiple oncogenes [23, 24]. Interestingly, when we digested freshly resected allografts into single cell suspensions and implanted them into immunocompetent C57BL/6J mice by multiple routes, we observed PCa metastasis in multiple soft tissues including lymph nodes, lungs, liver and spleen. Surprisingly, intravenous and intracardiac injections of B6CaP cells resulted in frequent skeletal metastasis to femur, vertebrae and skull. Further, the bone metastases generated frequent osteoblastic and osteolytic lesions. These observations indicate that B6CaP closely recapitulates clinical context. Thus, B6CaP line may serve as a valuable resource to understand the molecular underpinnings of skeletal metastasis.

Taken together, B6CaP line overcomes two critical limitations to study the skeletal metastasis of prostate cancer. First, unlike most other mouse models, B6CaP line metastasizes to bones, making it possible to study the mechanisms responsible for skeletal metastasis. The ability of B6CaP to bones also may allow researchers to dissect the tumor-bone microenvironment in greater detail. Second, B6CaP metastasizes in an immune-competent mouse, making it possible for researchers to study tumor-immune interactions and to investigate new interventions based on immunotherapy. Thus, B6CaP closely recapitulates the clinical observations, and may serve as a valuable to tool to understand the complexity of skeletal metastasis of prostate cancer in an immune competent environment.

## MATERIALS AND METHODS

### Mice

All experimental procedures were approved by the Johns Hopkins Institutional Animal Care and Use Committee (IACUC). Wild type C57BL/6J were obtained from Jackson Laboratories (Bar Harbor, ME, Stock 664). FVB-Tg (ARR2/Pbsn-MYC) 7Key (Hi-Myc, Strain 01XF5) mice were obtained from NCI Mouse Repository (Frederick, MD). Genotyping was performed using primer sets and protocols recommended by the vendor. Genomic DNA for PCR was isolated from tails. SNP analysis was performed by Jackson Laboratory Genome Scanning Services on tail biopsies from two founder B6. HiMyc males at >N10 generation. This 148 SNP panel can distinguish C57BL/6J and FVB/NJ and covers 19 autosomes and X chromosome with a density of 15–20 MB.

### Allograft establishment and passage

Allograft tumors were minced and dissociated in DMEM/F12 media containing 10% Fetal Bovine Serum (FBS), Collagenase/Hyaluronidase mix (Stem Cell Technologies) at 37°C for 1 hour. 2.5 U/mL Dispase and 0.05 mg/ml DNAse I were added and cells were vigorously triturated for 1 min and passed through a 40 µm cell strainer with the aid of a syringe plunger. Cells were pelleted, washed twice, and resuspended in PBS. Live cells were counted with Trypan Blue dye and 1 × 10^6 viable cells in 100 µL PBS were injected subcutaneously in the shaved flank of C57BL/6J mice. Subcutaneous tumor size was measured with calipers. For intracardiac or intravenous inoculation, cells were similarly dissociated, and 1 × 10^5 viable cells in 100 µL PBS were injected via tail vein or by percutaneous intracardiac injection. For orthotopic inoculation, 1 × 10^5 cells in10 µL PBS were surgically injected into the anterior prostate lobe. To generate castration resistant cell lines, mice were surgically castrated when subcutaneous tumors measured 1 cm in greatest dimension. After complete regression, tumors were monitored weekly until regrowth began, which was considered castrate resistant recurrence.

### Organoid and cell culture

For organoid culture, dissociated cells were dissociated and cultured using established protocols [25]. In brief, dissociated cells were mixed with a 1:1 mixture of matrigel and organoid medium and pipetted around the rim of ultra-low attachment cell culture plates (Corning). After incubation at 37°C for 30 minutes, organoid medium was added to each well. Organoid medium consisted of Advanced DMEM/F-12 (Gibco) supplemented with 5% FBS, 10 ng/mL epidermal growth factor, and 10 µM Y-27632 (STEMCELL Technologies); 1X B-27, 2 mM HEPES, 1X Penicillin/Streptomycin (Gibco); and 10 nM DHT (Sigma). MyC-CaP cells were purchased from ATCC (CRL-3255) and cultured according to ATCC recommendations.

### Histology and immunohistochemistry

For histology, soft tissue was fixed in formalin and skull, femurs, and spine were decalcified using Formical-4 (StatLab). Tissues were routinely processed, sectioned, and stained with hematoxylin and eosin (H&E). For immunohistochemistry, slides were deparaffinized and rehydrated before steaming in EDTA HIER Buffer (Thermo Scientific) for 20 minutes. Endogenous peroxidases were quenched with BLOXALL (Vector Labs), and sections blocked with Normal Horse Serum (Vector Labs). Slides were incubated with antibodies directed against CK8 (Covance), AR (Santa Cruz), MYC (Abcam), E-cadherin (Cell Signaling), CK14 (Covance), Synaptophysin (Abcam), IBA-1 (Wako Chemical), Pancytokeratin (Sigma), Ki67 (Abcam), F4/80 (Cell Signaling) and CD11b (Abcam). Staining was visualized with ImmPRESS HRP Polymer detection kit and ImmPACT DAB (Vector Labs). After staining, slides were scored 0–4 based on number of positive cells and intensity of staining.

### Flow cytometry

In order to obtain tumor infiltrating lymphocytes, tumors from third passage B6CaP cells grown in parallel were mechanically disintegrated and digested for 1 h at 37°C in RPMI media with 10 mg/ml of Collagenase IV and 1 mg/ml of DNase. After digestion, white blood cells were isolated from tumors using anti CD45 antibodies conjugated with magnetic beads (Miltenyi Biotec, Bergisch Gladbach, Germany) according to manufacturer’s protocol. Tumor infiltrating leukocytes were characterized with antibodies against CD3 (clone 145-2C11, eBioscience), CD19 (clone 1D3, BD Biosciences), CD49b (clone DX5, BD Biosciences), CD4 (clone H129.19, BD Biosciences), CD8 (clone 53-6.7, BD Biosciences), PD-1 (clone J43, eBioscience), Tim-3 (clone RMT3-23, eBioscience), Lag-3 (clone C9B7W, eBioscience) and CTLA4 (clone UC10-4F10-11, BD Biosciences). Flow through cells not captured by bead selection were stained with PDL-1 antibody (clone MIH5, eBioscience). For viability staining we used Live/Dead Aqua (Life Technologies). Gating controls were prepared with Fluorescence Minus One (FMO) stains of splenocytes or isotype control for tumor cells. Data were analyzed with FlowJo software (TreestarInc, Ashland, OR, USA).

### Statistical analysis

For comparisons between two groups, *p* values were calculated using two-tailed Student’s *t*-test in Prism GraphPad Software. Differences were considered statistically significant for *p* < 0.05.

## SUPPLEMENTARY MATERIALS




