# Supplementary material for: A mouse model of prostate cancer bone metastasis in a syngeneic immunocompetent host
Source: Oncotarget. 2019 Dec 3;10(64):6845–54. doi: 10.18632/oncotarget.27317 (PMC6901336; doi:10.18632/oncotarget.27317)
Supplement: Supplementary file 1 [file oncotarget-10-6845-s001.pdf]

A mouse model of prostate cancer bone metastasis in a syngeneic immunocompetent host

SUPPLEMENTARY MATERIALS

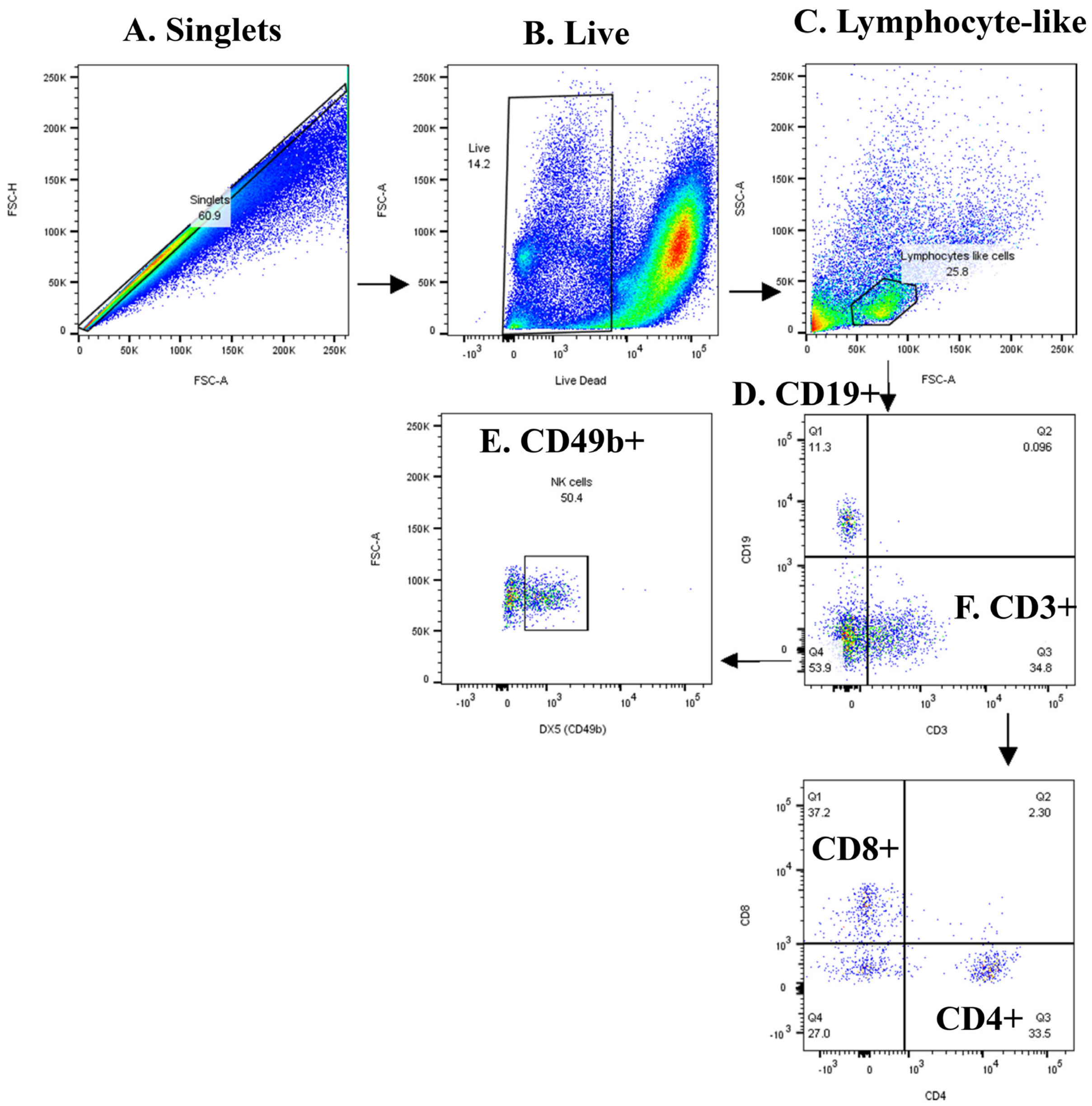

Supplementary Figure 1: Representative flow cytometry gating strategy for tumor infiltrating lymphocytes after magnetic bead enrichment of CD45+ cells from dissociated subcutaneous B6CaP allografts. (**A**) Selection for singlets; (**B**) selection for live cells; (**C**) selection for lymphocyte-like cells based on FSC/SSC. (**D**) Identification of CD3- CD19+ B cells (**E**) Identification of CD19- CD3- CD49+ NK cells. (**F**) Separation of CD3+ T cells by CD4/CD8 expression.

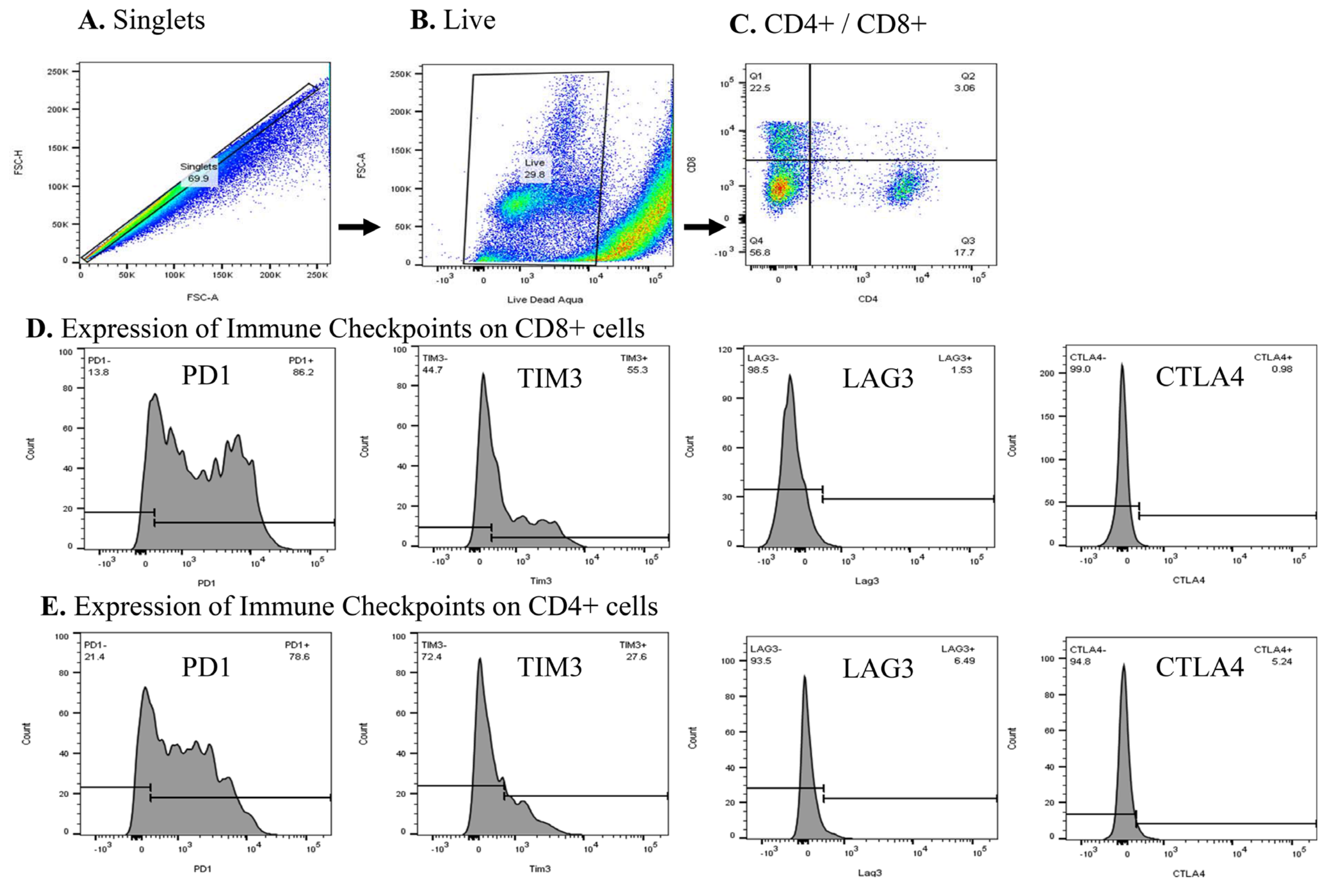

Supplementary Figure 2: Representative flow cytometry gating strategy for immune checkpoint expression on tumor infiltrating lymphocytes after magnetic bead enrichment of CD45+ cells from dissociated subcutaneous B6CaP allografts. (**A**) Selection for singlets; (**B**) selection for live cells; (**C**) selection for CD4/CD8 positive cells; (**D**, **E**) histogram evaluation of PD1, TIM3, LAG3, and CTLA4 in CD8+ cells (D) or CD4+ cells (E).

Supplementary Table 1: SNP analysis of B6-HiMyc model. See Supplementary Table 1
